# Integrated metabolomics and transcriptomics reveal the anti-aging effect of melanin from *Sepiella maindroni* ink (MSMI) on D-galactose-induced aging mice

**DOI:** 10.18632/aging.202890

**Published:** 2021-04-21

**Authors:** Yueyue Zhou, Weiwei Song, Chunlin Wang, Changkao Mu, Ronghua Li

**Affiliations:** 1 Key Laboratory of Applied Marine Biotechnology, Ministry of Education, Ningbo University, Ningbo 315211, China; 2 Collaborative Innovation Center for Zhejiang Marine High-Efficiency and Healthy Aquaculture, Ningbo University, Ningbo 315211, China

**Keywords:** *Sepiella maindroni*, melanin, antiaging, metabolomics, cDNA microarray

## Abstract

*Sepiella maindroni* ink, a flavoring and coloring agent in food, has attracted considerable attention due to its various pharmacological activities. Our previous study showed that the melanin of *Sepiella maindroni* ink (MSMI) can alleviate oxidative damage and delay aging in D-galactose(D-gal)-induced aging mice. This study aimed to reveal the possible mechanisms of the anti-aging effect of MSMI. In this article, a comprehensive analysis of gas chromatography-mass spectrometry (GC-MS)-based metabolomics and microarray-based transcriptomics revealed that 221 mRNAs were differentially expressed and 46 metabolites were significantly changed in the anti-aging progress of MSMI. Integrated analysis of transcript and metabolic profiles indicated that MSMI mainly altered carbohydrate metabolism, lipid metabolism, and insulin signaling pathway. MSMI achieved anti-aging effects not only by reducing oxidative damage and sorbitol toxicity but also by regulating lipid metabolism, improving insulin sensitivity, and reducing the formation of advanced glycation end products (AGEs). Moreover, our findings firstly demonstrated that MSMI could increase the expression of interferon-induced proteins and might be a potential antiviral compound.

## INTRODUCTION

Cuttlefish ink, a byproduct of seafood processing, has been used as a traditional Chinese medicine, possessing antibacterial and antitumor activities [1]. The major component of cuttlefish ink is melanin, a macromolecule formed by the oxidative polymerization of phenolic or indolic compounds. Melanin manifests diverse biological and pharmacological properties, including photoprotection, antiradiation, and antioxidation [2]. Besides, recent research has revealed other functions of melanin, including that of anti-cancer [3], protecting the liver, and regulating gastrointestinal health [4]. *Sepiella maindroni* is well-known as one of the most popular dietary cephalopod species in the coastal area of China. MSMI is the most prevalent melanin because of its large abundance, low cost, and easy extraction. In our previous study, we demonstrated that MSMI effectively reduced oxidative stress damage in aging mice induced by D-gal [5]. Besides, Han et al. investigated the effect of MSJI (natural melanin extracted from *Sepiella japonica* ink) treatment on the expression of miRNAs. They identified eight differentially expressed miRNAs associated with reducing oxidative damage [6]. Overall, previous studies revealed that MSMI could alleviate oxidative damage and delay aging in D-gal-induced aging mice. However, the anti-aging mechanisms of MSMI and its effect on metabolic and gene expression profiles remain unclear.

The aging process is a universal, intrinsic, progressive accumulation of deleterious changes in tissues and organs [7, 8]. In recent years, the development of anti-aging agents has become a research hotspot. The D-gal-induced aging model mice have been widely used in pharmacological studies of anti-aging agents because this animal model exhibits many symptoms that resemble accelerated aging [9]. In this research, we studied the anti-aging mechanisms of MSMI in D-gal-induced aging model mice.

Metabolomics is a powerful systemic approach to detect subtle metabolic changes in tissues and biological fluids caused by stimuli such as drugs, genetic effects, and disease processes [10]. Meanwhile, cDNA microarray is a popular tool for investigating the expression levels of thousands of genes simultaneously [11]. It has been proved that the aging process is accompanied by disturbances in gene expression and metabolism [12]. Therefore, to better understand the anti-aging effect of MSMI, we used the GC/MS-based metabolomics and microarray-based transcriptomics to comprehensively evaluate the changes in transcript and metabolic profiles and reveal the potential molecular mechanisms underlying the anti-aging ability of MSMI.

## RESULTS

### MSMI-induced changes in gene expression

Our previous study has indicated that MSMI could reduce oxidative damage and delay aging [5]. In this study, to further explore the anti-aging mechanisms of MSMI, we performed metabolomics and transcriptomics analyses on liver samples isolated from control group, D-gal aging model group (DM group), and high MSMI treated group (MT group). Firstly, we performed gene expression analysis using cDNA microarray.

**Figure 1:**
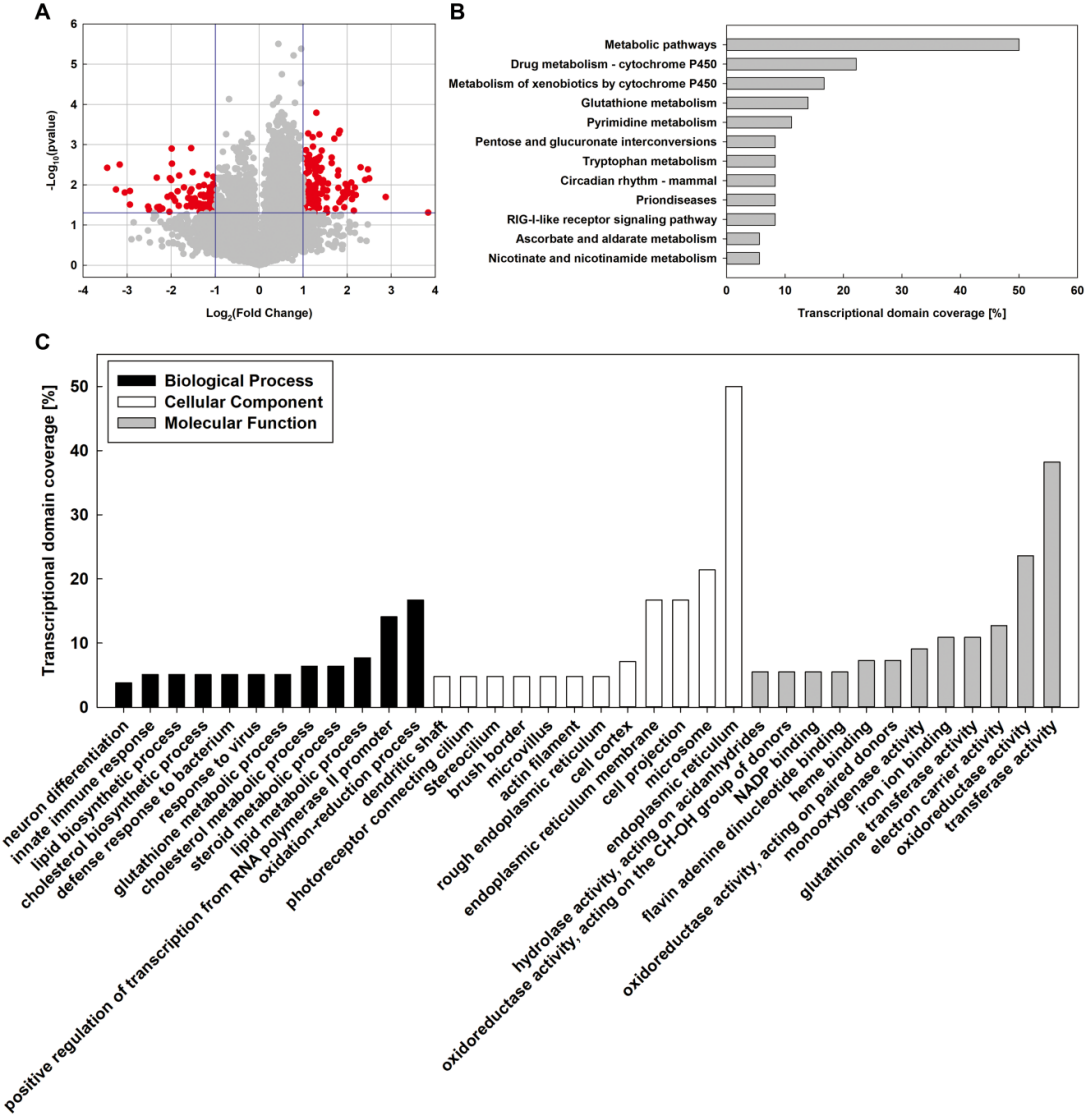
**Whole-genome microarray analysis of MT and DM groups.** (**A**) Volcano plot analysis of mRNA expression variation between MD and MT groups. (**B**) KEGG pathway analysis was used to identify key pathways and biological functions. (**C**) Gene Ontology (GO) analysis of differentially expressed genes (DEGs). DEGs are classified into three major domains: biological process (BP), cellular component (CC) and molecular function (MF).

Compared with DM group, 2801 mRNAs were found to be differentially expressed in MT group (*p*-value < 0.05). Volcano plot filtering was used to identify genes that were differentially expressed with statistical significance between the MT and DM groups (fold change ≥ 2.0 and *p*-value < 0.05) (Figure 1A). A total of 221 mRNAs, including 133 up-regulated mRNAs and 88 down-regulated mRNAs, were significantly changed in MT group (fold change ≥ 2.0 and *p*-value < 0.05). The differentially expressed mRNAs in MT group are listed in Supplementary Table 1.

To verify the results of the cDNA microarray, six differentially expressed genes were selected randomly for validation by quantitative real-time PCR (qRT-PCR). *Ces2a*, *Cyp1a2*, *Fmo3*, and *Alas1* were significantly up-regulated, meanwhile *Prom1* and *Derl3* were significantly down-regulated in MT group compared with DM group (Supplementary Figure 1). Overall, the qRT-PCR results showed that the expression patterns of qRT-PCR were consistent with those of cDNA microarray.

To investigate the possible biological functions of the genes differentially expressed in MT group, all differentially expressed genes (DEGs) were further analyzed by Gene Ontology (GO) (Figure 1C). In the biological process category, 16.7% of the genes were associated with oxidation-reduction process followed by positive regulation of transcription from RNA polymerase II promoter (14.1%), lipid metabolic process (7.7%), steroid metabolic process (6.4%), and cholesterol metabolic process (6.4%). Under the cellular component category, the majority of genes were involved in endoplasmic reticulum (50.0%) and microsome (21.4%) followed by cell projection (16.7%) and endoplasmic reticulum membrane (16.7%). Within the molecular function category, highly represented genes belonged to the transferase activity (38.2%), oxidoreductase activity (23.6%), electron carrier activity (12.7%), and glutathione transferase activity (10.9%). Moreover, the Kyoto Encyclopedia of Genes and Genomes (KEGG) pathway significant enrichment analysis revealed the distinct gene networks in the liver affected by MSMI feeding. The DEGs were involved in 14 pathways, mainly related to metabolic pathways (50%), drug metabolism-cytochrome P450 (22.2%), metabolism of xenobiotics by cytochrome P450 (16.7%), and glutathione metabolism (13.9%). The pathways with the greatest number of sequences are shown in Figure 1B.

Overall, the transcription levels of 221 genes were significantly changed in MT group, with 133 genes being upregulated and 88 being downregulated. Based on GO analysis, 16.7% of the genes were associated with oxidation-reduction. Based on KEGG analysis, 13.9% of the genes were associated with glutathione metabolism. Besides, 20.5% of the genes were associated with lipid metabolism (7.7% related to lipid metabolic process, 6.4% related to steroid metabolic process, and 6.4% related to cholesterol metabolic process). Considering that oxidative damage and lipid metabolism disorder are important factors leading to aging [9], MSMI might reduce oxidative damage and regulate lipid metabolism to achieve the anti-aging effect.

### MSMI-induced changes in metabolic profiles

To determine whether MSMI influences metabolic profiles and identify the differential metabolites (DMs), the principal component analysis (PCA), the partial least squares discriminant analysis (PLS-DA), and the orthogonal projections to latent structures-discriminant analysis (OPLS-DA) were performed between DM and MT groups following GC/MS data analysis.

**Figure 2:**
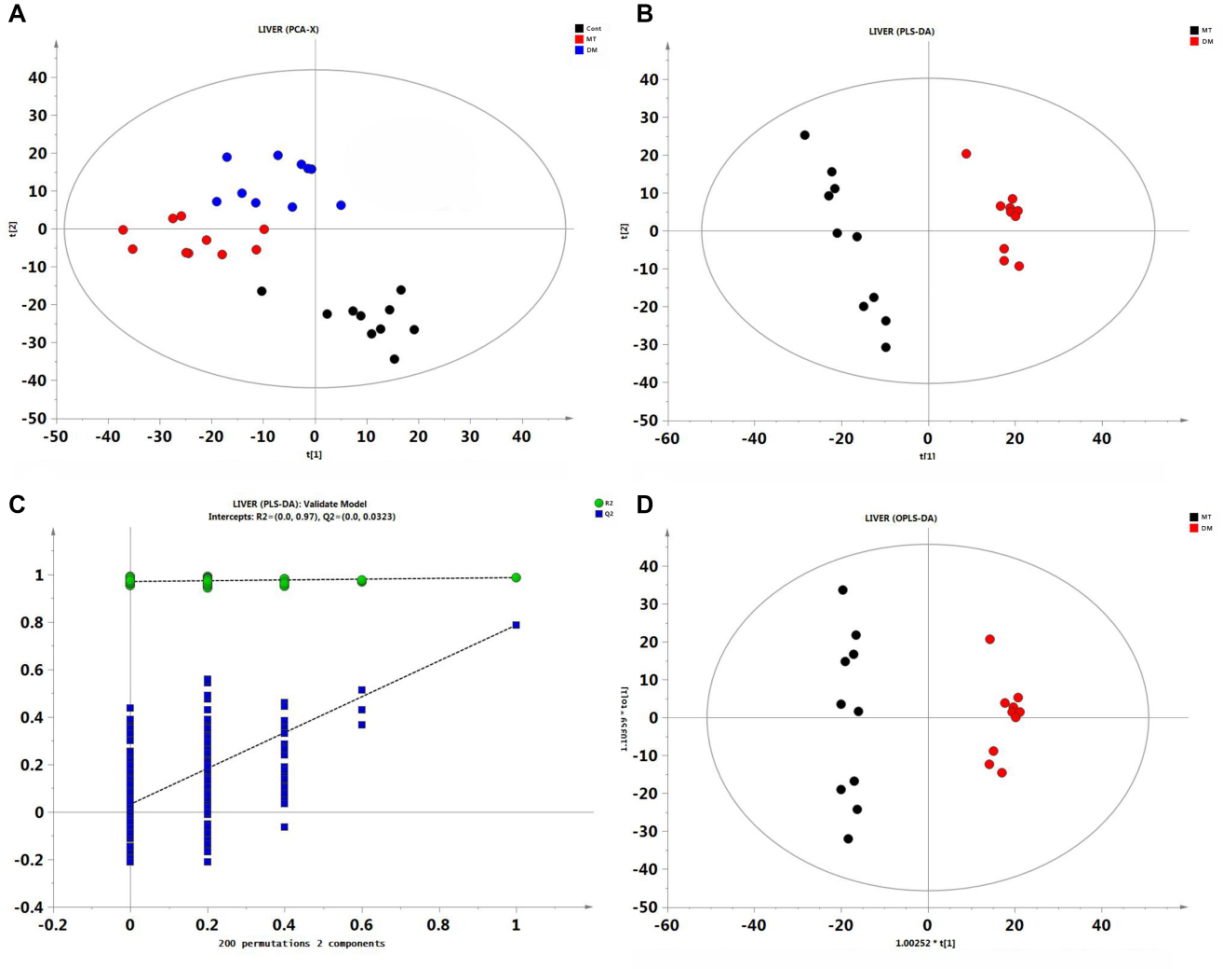
**Multivariate statistical analysis of liver GC/MS data.** (**A**) PCA score plot among control, DM and MT groups. (**B**) PLS-DA score plot comparing DM and MT groups. (**C**) The result of permutation test, R2 (green circle), Q2 (blue square). (**D**) PLS-DA score plot comparing DM and MT groups.

Firstly, to obtain general metabolic trends, PCA was performed on the GC/MS data. Parameters collected from control, DM, and MT groups were separated by PCA and data were in 95% confidence intervals (Figure 2A). There was an obvious separation between control and DM groups, indicating that D-gal in the DM group caused significant changes in the metabolic profile compared with control group. Furthermore, MT group was separated from control group and DM group, suggesting that MSMI could alter the metabolic profile of D-gal-induced aging mice.

Following PCA, PLS-DA model was constructed to better distinguish DM and MT groups. Clear separation between DM and MT groups was observed in the PLS-DA score plots (Figure 2B), indicating that the metabolic profile was significantly changed in MT group compared with DM group. A permutation test was performed to further verify the PLS-DA model (Figure 2C). R2 and Q2 intercept values were (0.0,0.970) and (0.0,0.0323), respectively. The lower Q2 intercept value indicated the high reliability and high stability of this model. As an extension of PLS-DA model, OPLS-DA model was used in this study to obtain the differential metabolites (DMs) between DM and MT groups. Based on the VIP (variable importance projection, VIP > 1.0) and *p*-value (*T*-test, *p* < 0.05), 46 metabolites were identified between DM and MT groups, including cholesterol, sorbitol, sophorose2, xylose1, and erythrose 2. Detailed information related to these metabolites can be found in Supplementary Table 2.

MSMI could alter the metabolic profile of DM group. The levels of 46 metabolites changed significantly in MT group compared with DM group. Among the differentially altered metabolites, the concentrations of cholesterol and sorbitol in MT group were significantly lower than that in DM group. The accumulation of sorbitol is known to be harmful to the body and makes a great contribution to the aging process [9, 13]. D-gal could cause lipid metabolism disorder and insulin resistance and eventually lead to body aging [14]. Cholesterol and sorbitol levels were significantly increased in D-gal-induced aging mice [9]. Compared with DM group, the concentrations of sorbitol and cholesterol in MT group were significantly reduced (Supplementary Table 2). The results indicated that MSMI might reduce sorbitol toxicity and regulate lipid metabolism to achieve the anti-aging effect. Moreover, compared with DM group, the levels of reducing sugars (sophorose2, xylose1, and erythrose 2) in MT group were significantly decreased. High concentrations of reducing sugars might lead to an increase of Maillard reaction and then increase the levels of AGEs, which could damage tissues and cause aging by crosslinking proteins and inducing inflammatory responses [15]. The contents of these three reducing sugars were significantly increased in D-gal aging mice, while MSMI feeding can reduce their contents. The results suggested that MSMI might achieve the anti-aging effect by blocking the formation of AGEs. Collectively, the anti-aging effect of MSMI might be achieved by reducing sorbitol toxicity, regulating lipid metabolism, and blocking the formation of AGEs.

### Integrated analysis of transcriptomic and metabolomic profiles

To better interpret results obtained from single metabolomic/transcriptomic data, we performed an integrated analysis of the transcriptomics and metabolomics datasets according to KEGG pathway database. The changes of KEGG pathways induced by MSMI are mainly related to carbohydrate metabolism, lipid metabolism, and insulin signaling pathway.

### Carbohydrate metabolism alterations

**Figure 3:**
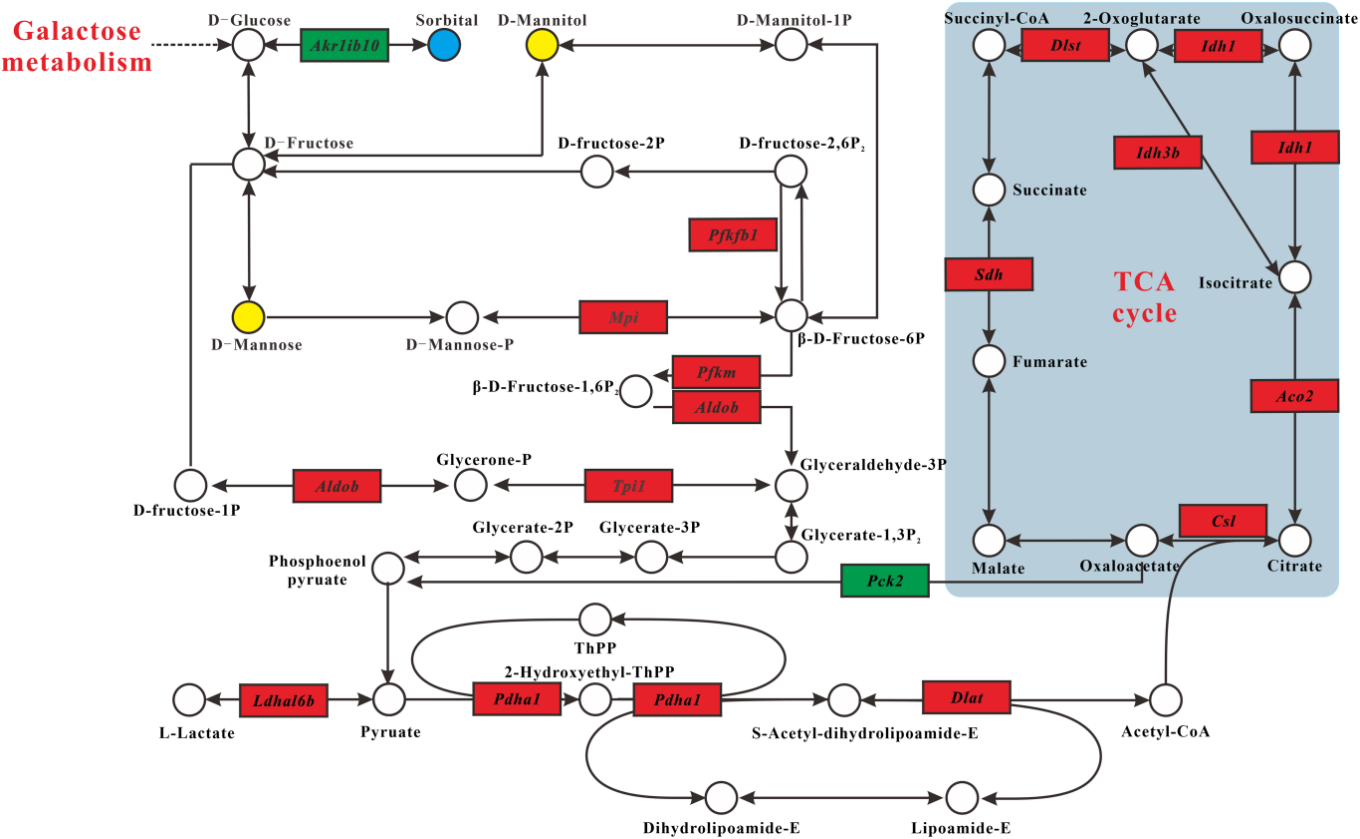
**Carbohydrate metabolism alterations.** Upregulated and downregulated genes in MT group compared with DM group are depicted as red box and green box espectively. Metabolites up- and downregulated are shown by yellow and blue circles, respectively.

Integration of the metabolomics and transcriptomics data demonstrated that MSMI could affect carbohydrate metabolism. Interconnected pathways included fructose and mannose metabolism pathway, glycolysis/gluconeogenesis pathway, and TCA cycle pathway (Figure 3). In general, MSMI could reduce the accumulation of sorbitol by decreasing the expression of *Akr1b10* gene and increasing the expression of *Pfkfb1*, *Mpi*, *Pfkm*, *Aldob*, and *Tpi1* genes. Moreover, MSMI could promote glycolysis through the upregulation of *Pdha1* and *Dld* genes, inhibit gluconeogenesis by inhibiting the expression of *Pck2*, and promote the TCA cycle through the upregulation of *Csl*, *Aco2*, *Idh1*, *Idh3b*, *Dlst*, and *Sdh* genes.

In fructose and mannose metabolism pathway (Figure 3), the concentration of sorbitol in MT group was significantly lower than that in DM group, while the concentrations of D-mannose and D-mannitol in MT group were higher than that in DM group. In addition, the expression of *Akr1b10* was significantly decreased in MT group compared with DM group. These results indicated that D-glucose generated by galactose metabolism was mainly converted to D-mannose and D-mannitol, rather than sorbitol. MSMI feeding also up-regulated the expression levels of *pfkfb1, Mpi, Pfkm, Aldob,* and *Tpi1* genes, which encode fructose-2, 6-bisphosphate-2-phosphatase, mannose-6-phosphate-isomerase, 6-phosphofructokinase, fructose-bisphosphate aldolase, and triose-phosphate isomerase, respectively (Figure 3). These five enzymes promoted the formation of β-D-Fructose-6P, β-D-Fructose-1,6P_2_, and glyceraldehyde-3P, which are the substrates for glycolysis. The transformation of D-mannose and D-mannitol to glycolysis might be accelerated by the up-regulation of these five genes.

In Glycolysis/Gluconeogenesis pathway, the expression levels of *Pdha1* and *Dld* genes, which encode pyruvate-dehydrogenase and dihydrolipoyl-dehydrogenase, respectively, were significantly increased in MT group compared with DM group (Figure 3). These two enzymes are parts of the mitochondrial pyruvate dehydrogenase complex (PDC), which catalyzes the oxidative decarboxylation of pyruvate to acetyl-CoA. MSMI might promote glycolysis through the high expression of these two genes. Moreover, the expression level of *Pck2* gene, which encodes the cytosolic isozyme of phosphoenolpyruvate carboxykinase (PEPCK), was decreased in MT group (Figure 3). PEPCK is a hub molecule linking the tricarboxylic acid (TCA) cycle, glycolysis, and gluconeogenesis in the liver [16]. Overexpression of PEPCK in the liver leads to hyperglycemia and type 2 diabetes [17]. MSMI might inhibit gluconeogenesis and decrease blood glucose by decreasing the expression of gluconeogenesis key rate-limiting enzymes of PEPCK.

In the TCA cycle pathway, the expression levels of several genes (*Csl*, *Aco2*, *Idh1*, *Idh3b*, *Dlst*, and *Sdh*) encoding enzymes involved in the TCA cycle in MT group were significantly greater than those of DM group. MSMI might promote the TCA cycle through the high expression of these six genes.

### Lipid metabolism alterations

**Figure 4:**
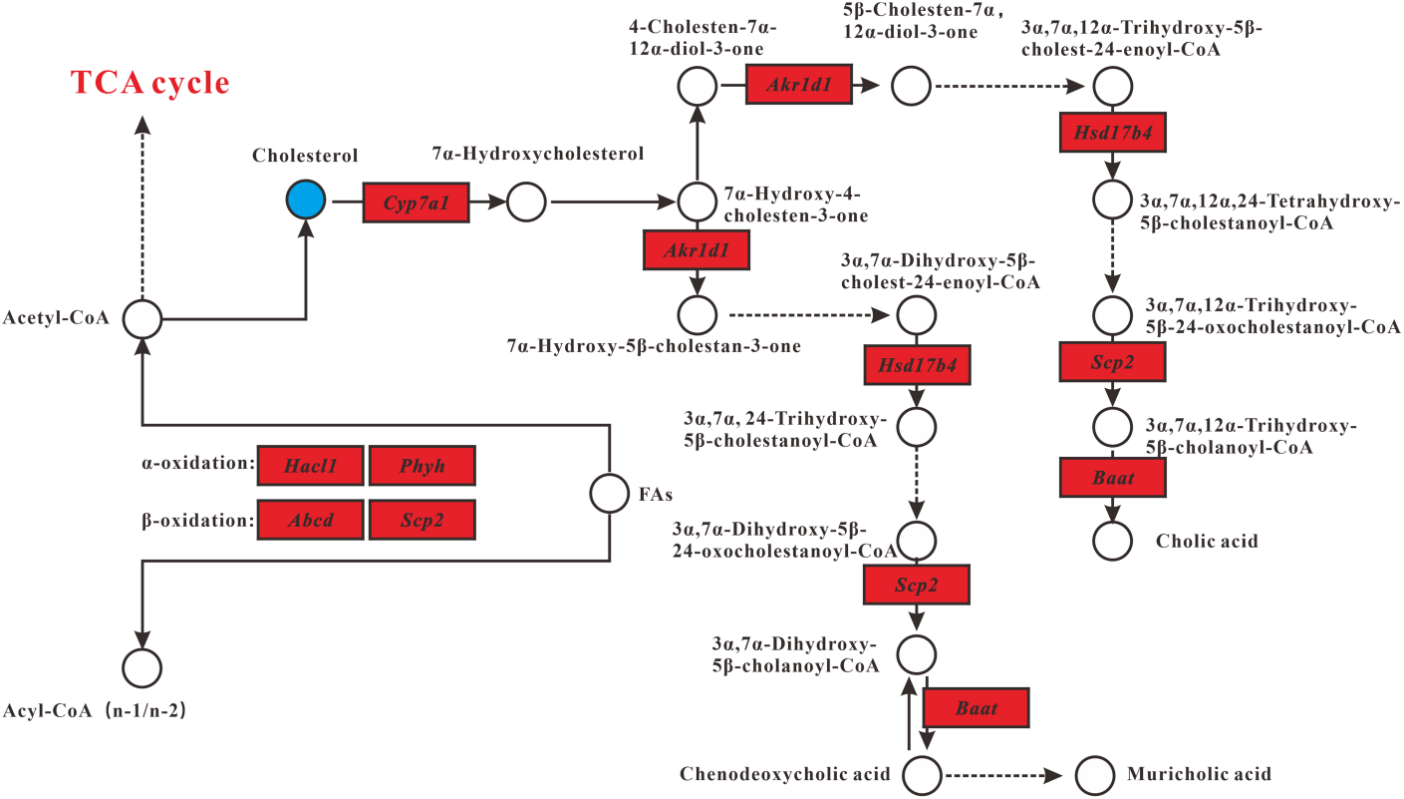
**Lipid metabolism alterations.** Upregulated and downregulated genes in MT group compared with DM group are depicted as red box and green box respectively. Metabolites up- and downregulated are shown by yellow and blue circles, respectively.

Combined analysis of metabolomics and transcriptomics revealed changes in the lipid metabolism pathway in MT group compared with DM group. Interrelated pathways included primary bile acid biosynthesis and fatty acid oxidation (Figure 4). In general, MSMI could accelerate the metabolism of cholesterol by the upregulation of *Akr1d1*, *Hsd17b4*, *Scp2*, and *Baat* genes. MSMI could promote fatty acid metabolism by the upregulation of *Hacl1*, *Phyh* and *Abcd* genes.

Compared with the control group, the cholesterol concentration in DM group was significantly increased, which is consistent with previous reports suggesting that D-gal could cause lipid metabolism disorders and eventually lead to body aging [9, 14]. However, compared with DM group, the cholesterol concentration in MT group was significantly reduced, suggesting that MSMI feeding could alleviate the lipid metabolism disorder caused by D-Gal (Figure 4).

Primary bile acid biosynthesis is the major cholesterol catabolic pathway. After synthesis, bile acids in bile are further concentrated in gallbladder and released into the small intestine postprandially [18]. Hepatocytes synthesize primary bile acids via two pathways, namely the classic pathway and the alternative pathway. It is believed that the classic bile acid synthesis pathway accounts for about 50% of the total bile acid production in mice [19]. In MT group, genes (*Akr1d1*, *Hsd17b4*, *Scp2*, and *Baat*) involved in the classical pathway were significantly upregulated, including the gene *Cyp7a1*that encodes cholesterol 7α-monooxygenase, the rate-limiting enzyme in the classical bile acid synthesis pathway (Figure 4).

In the fatty acid oxidation pathway, compared with DM group, the expression levels of several genes (Hacl1, Phyh, and Scp2) encoding enzymes involved in β-oxidation and α-oxidation were increased in MT group (Figure 4). Besides, the expression of Abcd, encoding the ABC transporter D, was up-regulated in MT group. ABC transporter D (ABCD) is very important for the oxidation of very-long-chain fatty acids (VLCFAs). VLCFAs enter the peroxisome as coenzyme A (CoA) esters via ABCD and subsequently undergo one or several rounds of β-oxidation [20].

### Changes in the insulin signaling pathway

Insulin, a hormone that regulates blood sugar, increases glucose uptake, and suppresses hepatic glucose production. Besides, the insulin/insulin-like growth factor signaling cascade also performs a broad range of functions, including the control of sugar, protein, and lipid metabolism as well as the regulation of growth [21]. MSMI might change insulin signaling, thereby affecting carbohydrate metabolism and lipid metabolism. In general, MSMI might promote glucose uptake by the upregulation of *Crk* gene, block insulin-stimulated lipogenesis by the downregulation of *Prkcz* gene, mediate enhanced lipolysis by the upregulation of *Prkar2a* and *Egr1* genes, inhibit gluconeogenesis by inhibiting the expression of *Pck2* gene, and enhance insulin sensitivity by inhibiting the expression of *Egr1* and *Prkcz* genes.

**Figure 5:**
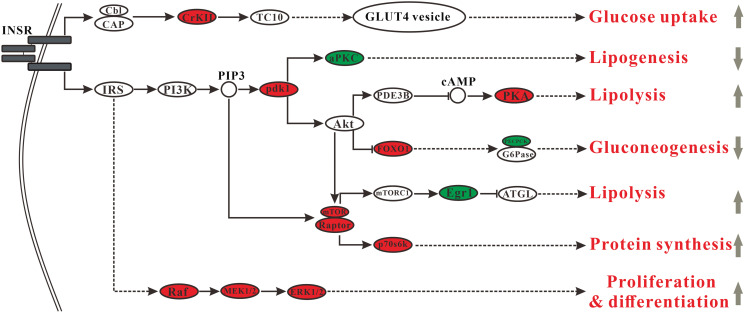
**Changes in the insulin signaling pathway.** Ellipses indicate those proteins involved in the insulin signaling pathway (red, upregulated proteins in MT group; green, downregulated proteins in MT group).

In the insulin signaling pathway, the expression of *Crk* gene, encoding adapter protein CrkII, was upregulated in MT group compared with DM group (Figure 5). CrkII is an important component in the CAP-Cbl-Tc10 pathway. Activation of CAP-Cbl-Tc10 is essential for GLUT4 translocation and glucose uptake [22]. Upregulation of *Crk* in MT group might increase the activity of GLUT4 translocation and then improve glucose uptake. Moreover, compared with DM group, the expression of *Prkcz* gene encoding atypical protein kinase C (aPKC), which mediates stimulatory effects of insulin on hepatic lipogenesis, was significantly down-regulated in MT group (Figure 5). It has been reported that excessive activity of hepatic aPKC seems to be a commonly observed and critically important contributor to insulin resistance in high-fat-fed (HFF) mice [23]. Downregulation of *Prkcz* in MT group might inhibit hepatic lipogenesis and improve insulin sensitivity. The gene *Prkar2a* encoding cAMP-dependent protein kinase A (PKA) was up-regulated in MT group compared with DM group (Figure 5). PKA mediates phosphorylation of lipolytic enzymes and lipolysis in a PI3K-PDE3B-cAMP pathway [24]. Upregulation of *Prkar2a* in MT group might activate PKA-induced lipolysis. The gene *Pck2* encoding phosphoenolpyruvate carboxykinase (PEPCK) was downregulated in MT group compared with DM group. Insulin suppresses hepatic gluconeogenesis by diminishing mRNAs encoding phosphoenolpyruvate carboxykinase (PEPCK) and glucose-6-phosphatase (G6Pase) [25]. MSMI might inhibit gluconeogenesis and decrease blood glucose by decreasing the expression of PEPCK, the rate-limiting enzyme of gluconeogenesis. The gene *Egr1* encoding early growth response 1(EGR1) was downregulated in MT group compared with DM group (Figure 5). mTORC1 suppresses lipolysis via Egr1-ATGL pathway. A high-fat diet could activate mTORC1, increase the levels of EGR1, and decrease ATGL expression [26]. An increase of EGR1 in adipose tissue is associated with insulin resistance and obesity. It also inhibits *Egr1* in adipocytes, which could effectively improve insulin sensitivity [27]. MSMI might promote lipolysis and enhance insulin sensitivity by inhibiting the expression of *Egr1* gene.

## DISCUSSION

D-gal aging model induces many changes that resembled natural aging progress and has been widely used for pharmacological studies of anti-aging agents. The aging effect of D-gal is mainly achieved through glucose and 1ipid metabolic disorders, oxidative damage, accumulation of AGEs, cell apoptosis, and insulin resistance [9, 14, 28]. Integrated analysis of transcript and metabolic profiles indicated MSMI could achieve the anti-aging effect not only by reducing oxidative damage and sorbitol toxicity but also by regulating lipid metabolism, improving insulin sensitivity, and reducing the formation of AGEs.

### MSMI achieves anti-aging effects by reducing oxidative damage and sorbitol toxicity caused by D-galactose

Three known pathways are involved in the degradation of galactose *in vivo* [29]. Many studies have reported that the galactitol (sorbitol isomer) produced in the second pathway and the peroxide accumulated in the third pathway are important factors that cause the aging of D-gal mice [9, 30].

MSMI could reduce oxidative damage caused by D-galactose. GO analysis showed that the significantly enriched biological process for MSMI feeding is oxidation-reduction process (16.7%). Our previous research showed that compared with DM group, MSMI could increase the activities of SOD, GSH-Px, CAT, and reduce MDA content in the mouse liver [5]. In this study, *Cat* and *sod2* genes encoding catalase (CAT) and superoxide dismutase (SOD) respectively, were up-regulated in MT group compared with DM group. In addition, MSMI significantly increased the expression of glutathione S-transferase superfamily genes (*Gstt3*, *Gsta3*, *Gstm1*, *Gstm2*, *Gstm3*, *Gstm4*). Glutathione S-transferases (GSTs) play a protective role against oxidative stress. GSTs are an important part of the cellular detoxification system, converting lipid peroxides and other peroxides into less harmful compounds [31]. These results indicated that MSMI might play an important role in alleviating the oxidative damage caused by D-gal. Taken together, MSMI might attenuate oxidative damage by the upregulation of *Cat*, *sod2*, and *GSTs* genes.

MSMI could reduce the content of sorbitol to achieve the anti-aging effect. The accumulation of sorbitol is known to be harmful to the body and makes a great contribution to the aging process of D-gal mice [9, 13]. The content of sorbitol (galactitol isomer) increased significantly in the D-galactose aging model. However, the content of sorbitol significantly decreased in MT group. As shown in carbohydrate metabolism (Figure 3), MSMI could reduce the accumulation of sorbitol by decreasing the expression of *Akr1b10* gene and increasing the expression of *Pfkfb1*, *Mpi*, *Pfkm*, *Aldob*, and *Tpi1* genes.

### MSMI achieves anti-aging effects by slowing lipid metabolism disorders

As a reducing sugar, D-gal inevitably causes lipid metabolism disorder and insulin resistance, finally leading to body aging [14]. Previous research has reported that D-gal facilitated the accumulation of cholesterol in the liver of mice [9, 32].

In this research, GO analysis showed that 20.5% of the genes were associated with lipid metabolism (7.7% related to lipid metabolic process, 6.4% related to steroid metabolic process, and 6.4% related to cholesterol metabolic process). In addition, metabolite analysis revealed that MSMI feeding could significantly reduce cholesterol content in the liver of MT group. Besides, as shown in lipid metabolism (Figure 4), MSMI could accelerate the metabolism of cholesterol by the upregulation of *Akr1d1*, *Hsd17b4*, *Scp2*, and *Baat* genes. MSMI could promote fatty acid metabolism by the upregulation of *Scp2,* *Hacl1*, *Phyh*, and *Abcd* genes. As shown in the insulin signaling pathway (Figure 5), MSMI might block insulin-stimulated lipogenesis by the downregulation of *Prkcz* gene. MSMI might mediate enhanced lipolysis by the upregulation of *Prkar2a* and *Egr1* genes. Overall, these results suggested that MSMI might achieve anti-aging effects by relieving lipid metabolism disorders caused by D-gal.

### MSMI achieves anti-aging effects by improving insulin sensitivity

Previous research has reported that D-gal regulated the ex pression of *Lepr* and *Egr1* genes, which could reduce insulin sensitivity, and produce insulin resistance [9, 32]. MSMI could target insulin sensitivity-related genes (*Prkcz, Egr1,* and *Il1b*) to improve insulin sensitivity. As shown in the insulin signaling pathway (Figure 5), MSMI might enhance insulin sensitivity by inhibiting the expression of *Egr1* and *Prkcz* genes. Also, interleukin 1 beta (IL1B), a pro-inflammatory pleiotropic cytokine, plays an important role in insulin resistance [27]. MSMI might improve insulin sensitivity by reducing IL1B expression in D-galactose aging mice. In addition, MSMI might alleviate lipid-induced insulin resistance by regulating lipid metabolism.

### MSMI achieves anti-aging effects by reducing the formation of glycosylated end products

D-gal induces the aging process, at least partially driven by the accumulation of AGEs [33]. Metabolomics analysis revealed that MSMI might block AGEs formation by reducing the content of reducing sugars (sophorose2, xylose1, and erythrose 2). The levels of reducing sugars (sophorose2, xylose1, and erythrose 2) in D-gal-induced aging model were significantly increased [9]. The levels of these three reducing sugars dropped significantly after MSMI feeding (Supplementary Table 2). High concentrations of reducing sugars could lead to an increase in Maillard reaction and then increase the levels of AGEs [15]. Gene expression profiling showed high transcription of *Dcxr* gene in MT group (Supplementary Table 1). Dicarbonyl/L-xylulose reductase (DCXR) plays an important role in removing α-dicarbonyl compounds (main precursors of AGEs) under oxidative conditions [34]. MSMI might inhibit AGEs precursor formation by overexpressing DCXR. Also, a recent study demonstrated MSMI could clear AGEs in serum [35]. Overall, MSMI could block AGEs formation by reducing the content of reducing sugars and downregulating *Dcxr* expression.

### MSMI treatment up-regulates antiviral gene expression

Surprisingly, compared with DM and control groups, several antiviral related genes (*Ifit3*, *Apol9b*, *Ifit1*, and *Isg15*) were highly expressed in MT group (Supplementary Table 1). IFIT1 and IFIT3 (encoded by *Ifit3* and *Ifit1*genes) are interferon-induced proteins with tetratricopeptide repeats (IFITs). IFITs is a family of IFN-stimulated antiviral proteins. IFIT1 binds tightly to non-self RNA, particularly capped transcripts lacking methylation on the first cap-proximal nucleotide, and inhibits their translation by out-competing the cellular translation initiation apparatus. Besides, IFIT3 is the central hub that enhances and regulates IFIT1 RNA binding [36]. Moreover, ISG15 (encoded by *Isg15* gene) is an interferon-induced ubiquitin-like modifier. ISG15 could be conjugated to various proteins, thus involved in a series of biological activity, encompassing antiviral defense, immune responses, and pregnancy [37]. Additionally, Apolipoprotein L9b (encoded by *Apol9b* gene) is an interferon-stimulated protein (ISG) that has antiviral activity. A previous report showed that *Apol9b* could inhibit the replication of Theiler's murine encephalomyelitis virus (TMEV) [38]. Only a few published studies reported the antiviral ability of melanin [39]. Our results suggested that MSMI could increase the expression of interferon-induced proteins and might serve as a potential antiviral compound worth exploring further.

## CONCLUSIONS

This study was designed to investigate the possible mechanisms of the anti-aging effect of MSMI, especially focusing on the changes of transcript and metabolic profiles. These results revealed that MSMI achieved anti-aging effects by reducing oxidative damage and sorbitol toxicity, regulating lipid metabolism, improving insulin sensitivity, and reducing the formation of AGEs. In addition, we found that MSMI could increase the expression of interferon-induced proteins and might be a potential antiviral compound. According to our results, MSMI could be applied as supplementary material in functional food products, especially for obese, diabetic, and aging people. The future research topic will focus on its application on other targets, to maximize its edible and pharmacological functions.

## MATERIALS AND METHODS

### Extraction of melanin from *Sepiella maindroni*

*Sepiella maindroni* was purchased from the Xiapu fish market (Xiapu, Fujian Province, China) with an average weight of 130 g and an average body length of 11cm. Melanin was prepared as previously described [40]. Briefly, the ink sacs of *Sepiella maindroni* were crushed to obtain ink, then the ink was filtered through the sterile cotton gauze to remove damaged tissue. Then, filtered ink was centrifuged at 8000 rpm for 10 minutes. After decanting the supernatant, the precipitations were freeze-dried to obtain crude melanin for further enzymatic treatment. Enzymatic hydrolysis was achieved by the addition of 1.5% alkaline protease, at 50 °C, pH 10.5, and the crude melanin concentration of 2% for 4 h. After hydrolysis, the reaction solution was centrifuged at 6000 rpm for 10 min. Then the supernatant was removed and the pellet was resuspended in 50 mL of ultrapure water (repeat six times). After the final wash, the precipitate was freeze-dried to obtain high-pure melanin.

### Animals and treatments

Sixty 6-week-old ICR mice used in this study were supplied by the Experimental Animal Center of Ningbo University (Ningbo, China). Mice were kept under constant temperature (24 ± 2°C) and humidity (60%) and were maintained on a reversed 12 h light: 12 h dark cycle. All experiments in this paper were conducted in compliance with the Chinese legislation regarding the use and care of laboratory animals and were approved by the Animal Care and Use Committee of Ningbo University.

After acclimatization to the laboratory environment, ICR mice were randomly divided into six groups (10 mice per group): control group, D-gal group, Vc group, MSMI treated groups of low (25 mg/kg/day), medium (120 mg/kg/day) and high (200 mg/kg/day) dosage. D-gal aging model group, Vc group (positive control), and MSMI treated groups were subcutaneously injected with D-gal (Sigma, St. Louis, MO, USA) at a dose of 120 mg/kg/day while the control group received the same volume of saline solution (0.9%). After subcutaneous injection, MSMI was orally administered at a dose of 25 mg/kg/day, 120 mg/kg/day or 200 mg/kg/day for MSMI treated groups. After a trial period of 45 days, all mice were sacrificed, and the serum, liver, brain, heart specimens were harvested and stored at ˗80°C for further experiments.

The content of malonaldehyde (MDA), the activities of catalase (CAT), superoxide dismutase (SOD), and glutathione peroxidase (GSH-px) of these six groups were measured [5]. Compared with the control group, the activities of CAT, SOD, and GSH-Px were markedly decreased in the D-gal aging model group. The improved activities of CAT, SOD,and GSH-Px were detected in Vc group and MSMI treated groups, with the most significant increase in the high MSMI dosage (200 mg/kg/day) group. Meanwhile, the content of MDA, which indirectly reflects the level of lipid peroxidation production, was significantly increased in the D-gal aging model group compared with control group, but these changes were remarkably intervened by MSMI, with the strongest intervention in the high dosage (200 mg/kg/day) group. These results indicated that MSMI could attenuate the increased oxidative damages in D-gal-treated mice and delay aging. The high dosage (200 mg/kg/day) group shows the best anti-aging effects [5].

In this study, to further explore the anti-aging mechanisms of MSMI, we selected control group, D-gal aging model group (DM group) and high MSMI treated group (MT group) as the research subjects to conduct the transcript and metabolic profiles studies. 30 liver samples from these three groups were used for metabolomics analysis and 9 of them were used for cDNA microarray analysis.

### Metabolite detection and identification

The liver tissue sample (100 mg) was transferred to a 2 mL microcentrifuge tube containing 50 μL L-2-chlorophenylalanine (0.1 mg/mL stock solution in dH2O; Sigma) and 0.5 ml extraction Solution (v_methanol_: v_chloroform_ = 3:1), and then homogenized the liver tissue in TissueLyser (Qiagen) at 70 Hz for 5 minutes. After homogenization, the samples were centrifuged at 4°C and 12000 rpm for 15 minutes. 0.4 mL of supernatant was transferred to a 2 mL glass autosampler vial and dried in a vacuum concentration dryer. Methoxyamination reagent (80 μL) (20 mg/mL in pyridine) was added to the glass autosampler vial and incubated at 37°C for 2 h. Subsequently, BSTFA regent (0.1 mL) (1% TMCS, v/v) was added to the glass autosampler vial and incubated at 70°C for 1 h. Samples were cooled to room temperature for further analysis.

The metabolites were identified by Agilent 7890 gas chromatography system (Agilent 7890A, Agilent, USA) coupled with Pegasus HT time-of-flight mass spectrometer (LECO Chroma TOF PEGASUS 4D, LECO, USA), as previously described [9]. Briefly, GC separation was performed on a DB-5MS capillary column (30 m × 250 μm inner diameter, 0.25-μm film thickness; J&W Scientific, Folsom, CA, USA). Helium was used as the carrier gas at a flow rate of 20 mL/min. Electron ionization mass spectrometry at full scan mode (m/z 85–600) was used for MS analysis.

### Whole-genome gene expression microarray assay

Total RNA was extracted from liver tissue using the mirVanaTM RNA isolation kit (Applied Biosystems, Darmstadt, Germany), and then purified using the Qiagen RNeasy Mini kit (Qiagen, Chatsworth, CA, USA). The total RNA was quantified using NanoDrop ND-2000 (Thermo Scientific, Wilmington, DE, USA) and the RNA integrity was evaluated using Agilent Bioanalyzer 2100 (Agilent Technologies, Palo Altlo, CA, USA). Subsequently, double-strand cDNA was synthesized using PrimeScript RT reagent Kit (TaKaRa BIO, Shiga, Japan) and then labeled with cyanine-3-CTP. The samples were microarray hybridized and washed using Gene Expression Hybridization Kit and Gene Expression Wash Pack (Agilent Technologies). The arrays were scanned by Agilent Scanner G2505C to generate array image files.

### qRT-PCR analysis

qRT-PCR was performed to investigate the changes in relative expression levels of genes (*Ces2a*, *Cyp1a2*, *Prom1*, *Fmo3*, *Alas1*, *Derl3*). Assays were performed using the QuantiTect SYBR Green PCR kit (Qiagen, Hilden, Germany) in an Eppendorf Realplex Real-Time PCR system (Eppendorf, Hamburg, Germany). Gene expression levels were normalized to GAPDH. The experiments were performed in duplicate with liver samples prepared from 3 animals per group. The fold changes of the selected genes were analyzed by the 2^˗ΔΔCT^ method [41]. qRT-PCR primers are shown in Supplementary Table 3.

### Data processing and analysis

Agilent Feature Extraction software (version 10.7.1.1) was used to analyze the acquired array images. Quantile normalization and subsequent data processing were performed using the GeneSpring version 12.5 software (Agilent Technologies). The microarray probes with at least 1 out of 2 conditions flagged in “P” were chosen for further data analysis. Differentially expressed genes with statistical significance were identified based on fold change ≥ 2.0 with *p*-value < 0.05 (*t*-test). Afterward, GO analysis and KEGG analysis were performed to determine the roles of these differentially expressed mRNAs.

The metabolic profiles of the samples were analyzed by multivariate statistical analysis, including the principal component analysis (PCA), the partial least squares discriminant analysis (PLS-DA), and the orthogonal projections to latent structures-discriminant analysis (OPLS-DA). Besides, SIMCA-P 13.0 software (Umetrics, Sweden) was employed as a multivariate analyzing tool. The robustness and predictive ability of PLS-DA model were evaluated by Leave-one-out validation (LOOCV). *T*-test (*p* < 0.05) combined with the VIP (Variable Importance in the Projection, VIP > 1.0) value of the first principal component in OPLS-DA model was used to identify differentially expressed metabolites.

Integrated enrichment analysis of transcriptomic and metabolomic data was conducted according to the Kyoto Encyclopedia of Genes and Genomes (KEGG) pathway database [42].

## Supplementary Materials

Supplementary Figure 1

Supplementary Tables
