# Supplementary material for: Integrated metabolomics and transcriptomics reveal the anti-aging effect of melanin from Sepiella maindroni ink (MSMI) on D-galactose-induced aging mice
Source: Aging (Albany NY). 2021 Apr 21;13(8):11889–906. doi: 10.18632/aging.202890 (PMC8109126; doi:10.18632/aging.202890)
Supplement: Supplementary Figure 1 [file aging-13-202890-s001.pdf]

SUPPLEMENTARY FIGURE

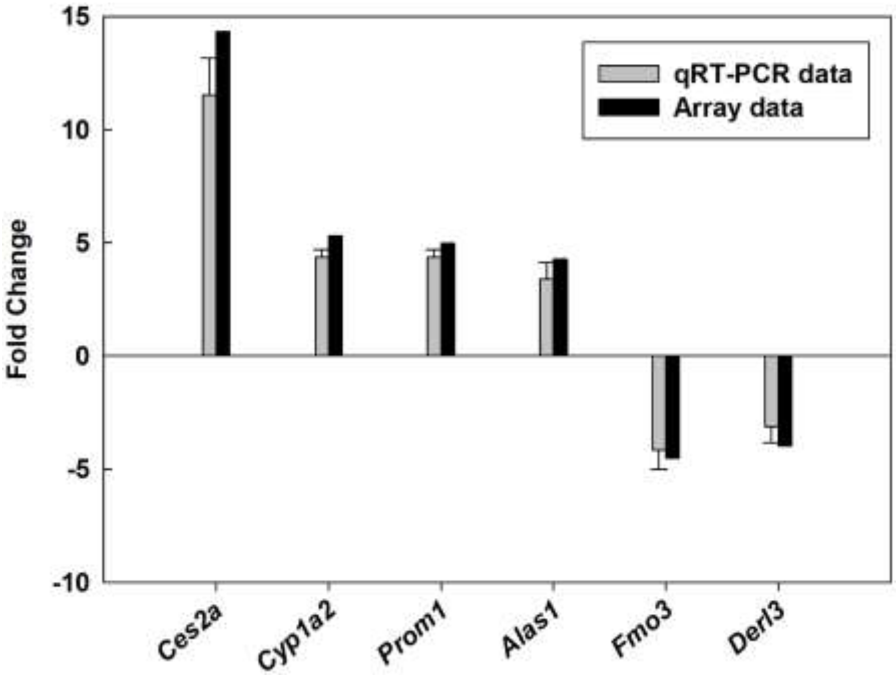

Supplementary Figure 1. Microarray validation by real-time PCR. The expression levels of genes *Ces2a*, *Cyp1a2*, *Fmo3*, *Alas1*, *Prom1*, and *Derl3* from gene array analysis compared with that of real-time PCR.
