# Supplementary material for: Integrated metabolomics and transcriptomics reveal the anti-aging effect of melanin from Sepiella maindroni ink (MSMI) on D-galactose-induced aging mice
Source: Aging (Albany NY). 2021 Apr 21;13(8):11889–906. doi: 10.18632/aging.202890 (PMC8109126; doi:10.18632/aging.202890)
Supplement: Supplementary Tables [file aging-13-202890-s002.pdf]

## SUPPLEMENTARY TABLES

**Supplementary Table 1. The differentially expressed mRNAs in MT group compared with DM group.**

| ProbeName | *p*-value | Fold Change | Regulation | Symbol | Gene.ID |
|---|---|---|---|---|---|
| A_55_P2293013 | 0.0493 | 14.3262 | up | *Ces2a* | 102022 |
| A_51_P327751 | 0.0070 | 5.6606 | up | *Ifit1* | 15957 |
| A_66_P128537 | 0.0042 | 5.5516 | up | *Isg15* | 100038880 |
| A_52_P595871 | 0.0076 | 5.3079 | up | *Cyp1a2* | 13077 |
| A_52_P402786 | 0.0365 | 4.9779 | down | *Prom1* | 19126 |
| A_51_P269404 | 0.0118 | 4.5178 | up | *Fmo3* | 14262 |
| A_55_P2083649 | 0.0088 | 4.2690 | up | *Alas1* | 11655 |
| A_51_P359570 | 0.0164 | 4.0861 | up | *Ifit3* | 15959 |
| A_51_P491667 | 0.0013 | 3.9660 | down | *Derl3* | 70377 |
| A_55_P2107775 | 0.0096 | 3.9027 | up | *Apol9a* | 223672 |
| A_51_P377856 | 0.0005 | 3.5075 | up | *Gstt3* | 103140 |
| A_55_P2102060 | 0.0139 | 2.9184 | up | *Gstm3* | 14864 |
| A_55_P1966438 | 0.0045 | 2.6146 | up | *Gstm2* | 14863 |
| A_51_P503625 | 0.0068 | 2.5512 | up | *Gsta3* | 14859 |
| A_65_P02177 | 0.0024 | 2.5473 | up | *Gstm4* | 14865 |
| A_55_P1966432 | 0.0068 | 2.4931 | up | *Gstm1* | 14862 |
| A_55_P2065231 | 0.0036 | 2.4913 | up | *Gstm3* | 14864 |
| A_51_P367866 | 0.0311 | 2.3314 | down | *Egr1* | 13653 |
| A_55_P2077628 | 0.0048 | 2.1923 | up | *Dcxr* | 67880 |
| A_55_P1961423 | 0.0126 | 2.1791 | up | *Gsta3* | 14859 |
| A_51_P284486 | 0.0022 | 2.0296 | up | *Gstm2* | 14863 |
| A_55_P2147136 | 0.0080 | 1.8937 | up | *Akr1d1* | 208665 |
| A_51_P138044 | 0.0199 | 1.8759 | up | *Foxo1* | 56458 |
| A_51_P132978 | 0.0223 | 1.7969 | up | *Idh1* | 15926 |
| A_55_P2002893 | 0.0346 | 1.7207 | up | *Pfkfb1* | 18639 |
| A_55_P2012974 | 0.0232 | 1.5369 | up | *Sdha* | 66945 |
| A_52_P200359 | 0.0064 | 1.5185 | up | *Aco2* | 11429 |
| A_51_P393897 | 0.0319 | 1.5009 | up | *Mpi* | 110119 |
| A_51_P335419 | 0.0050 | 1.4977 | up | *Csl* | 71832 |
| A_55_P1953980 | 0.0411 | 1.4949 | up | *Sdhb* | 67680 |
| A_51_P337269 | 0.0449 | 1.4874 | up | *Aldob* | 230163 |
| A_51_P290139 | 0.0099 | 1.4839 | up | *Dlst* | 78920 |
| A_55_P2146780 | 0.0161 | 1.4751 | up | *Sdhc* | 66052 |
| A_51_P130110 | 0.0360 | 1.4560 | up | *Idh3b* | 170718 |
| A_51_P184284 | 0.0106 | 1.4417 | up | *Dld* | 13382 |
| A_51_P445662 | 0.0120 | 1.4218 | up | *Hsd17b4* | 15488 |
| A_52_P43150 | 0.0152 | 1.3433 | up | *Baat* | 12012 |
| A_55_P1988424 | 0.0202 | 1.3282 | up | *Tpi1* | 21991 |
| A_51_P172573 | 0.0293 | 1.3242 | up | *Sod2* | 20656 |
| A_52_P600822 | 0.2236 | 1.3220 | up | *Prkcz* | 18762 |
| A_51_P409893 | 0.0221 | 1.3214 | up | *Prkar2a* | 19087 |
| A_55_P1970670 | 0.0309 | 1.2969 | up | *Scp2* | 20280 |
| A_51_P276063 | 0.0318 | 1.2544 | up | *Phyh* | 16922 |
| A_55_P2148873 | 0.0336 | 1.2515 | up | *Cat* | 12359 |
| A_51_P117995 | 0.0340 | 1.2481 | up | *Pfkm* | 18642 |
| A_51_P110888 | 0.0490 | 1.2452 | down | *Pck2* | 74551 |

The differentially expressed mRNAs discussed in this study are listed in the table. The full list of 2801 differentially expressed mRNAs can be obtained from the contact author.

**Supplementary Table 2. Metabolites detected and identified by GC/MS in MT group compared with DM group. Metabolites which VIP > 1 and *p*-value < 0.05 have been shown in Supplementary Table 2. If the value of log2 fold change is positive, the content of that metabolite in MT group is greater than DM group, If the value of log2 fold change is negative, the content of that metabolite in MT group is less than DM group.**

| Metabolites | RetentionTime | m/z | VIP | *p*-value | Fold change | log2 fold change |
|---|---|---|---|---|---|---|
| (-)-Dihydrocarveol | 10.7197 | 180 | 2.781200 | 0.015461 | 0.195113 | –2.357615 |
| 1-Kestose | 29.3104 | 204 | 1.557960 | 0.020102 | 3.066278 | 1.616489 |
| 21-hydroxypregnenolone 1 | 28.0997 | 243 | 3.370050 | 0.000286 | 0.215912 | –2.211485 |
| 2-amino-2-methylpropane-1,3-diol 1 | 10.7711 | 155 | 2.572560 | 0.027227 | 0.183154 | –2.448871 |
| 2'-deoxyadenosine 5'-monophosphate | 27.3751 | 169 | 2.798090 | 0.005003 | 0.378206 | –1.402758 |
| 3-hydroxybenzyl alcohol | 12.9109 | 135 | 3.799540 | 0.001584 | 0.236527 | –2.079921 |
| 4-Androsten-11beta-ol-3,17-dione 4 | 26.6908 | 259 | 1.687070 | 0.013457 | 0.280722 | –1.832788 |
| 4-hydroxyphenylacetic acid | 15.0040 | 177 | 2.454940 | 0.034226 | 0.173412 | –2.527727 |
| 4-Methylbenzyl alcohol | 10.1250 | 130 | 2.721800 | 0.002808 | 0.000003 | –18.448369 |
| Acetol 4 | 15.6426 | 97 | 1.614060 | 0.006122 | 0.382779 | –1.385416 |
| alpha-Santonin 1 | 21.1897 | 105 | 3.031090 | 0.004159 | 1.65E+06 | 20.655095 |
| Aminomalonic acid | 12.9258 | 232 | 1.015300 | 0.004899 | 0.624170 | –0.679990 |
| Anandamide 2 | 25.9614 | 91 | 1.117360 | 0.006817 | 0.495450 | –1.013190 |
| androsterone 1 | 23.7097 | 91 | 3.305390 | 0.004738 | 0.234040 | –2.095171 |
| asparagine 3 | 14.7457 | 244 | 1.961870 | 0.003549 | 0.462710 | –1.111818 |
| beta-hydroxypyruvate | 10.9797 | 260 | 2.313570 | 0.015057 | 0.320784 | –1.640327 |
| D-(glycerol 1-phosphate) | 16.3312 | 266 | 1.592740 | 0.037797 | 0.613434 | –0.705020 |
| D-galacturonic acid 2 | 18.2821 | 299 | 1.537300 | 0.044932 | 0.621460 | –0.686267 |
| Digitoxose 2 | 14.5501 | 244 | 2.439590 | 0.031031 | 0.258178 | –1.953560 |
| dihydrocoumarin 1 | 15.5767 | 217 | 2.377920 | 0.008405 | 22.517975 | 4.493005 |
| D-Talose 2 | 17.9603 | 211 | 2.120540 | 0.007450 | 4.407770 | 2.140049 |
| flavin adenine degrad product | 16.0677 | 191 | 3.786310 | 0.001679 | 0.202274 | –2.305614 |
| Gluconic lactone 1 | 17.5683 | 204 | 3.352460 | 0.016084 | 4.226876 | 2.079592 |
| glutamic acid | 14.7319 | 257 | 1.025780 | 0.025270 | 0.403922 | –1.307851 |
| glutaraldehyde 3 | 10.4367 | 85 | 2.147390 | 0.033205 | 3.115126 | 1.639291 |
| glycolic acid | 7.4198 | 103 | 1.078320 | 0.048699 | 0.571583 | –0.806966 |
| histidine 2 | 18.0133 | 154 | 2.437710 | 0.024060 | 0.436661 | –1.195415 |
| hydrocinnamic acid | 12.3431 | 104 | 2.377290 | 0.001093 | 3.291242 | 1.718632 |
| mannitol | 18.0940 | 304 | 1.196990 | 0.017301 | 2.132601 | 1.092614 |
| mannose 2 | 17.8777 | 367 | 3.113380 | 0.005807 | 2.70E+06 | 21.363729 |
| methyl trans-cinnamate | 12.1706 | 103 | 1.533310 | 0.001448 | 2.113743 | 1.079800 |
| N-Acetyl-L-leucine 2 | 12.6438 | 156 | 1.906730 | 0.041585 | 0.535818 | –0.900186 |
| N-Methyl-L-glutamic acid 3 | 12.8749 | 98 | 1.102860 | 0.001963 | 0.559949 | –0.836631 |
| Octadecanol | 20.2316 | 328 | 1.522260 | 0.014993 | 2.314538 | 1.210724 |
| oxalic acid | 8.1371 | 190 | 3.197850 | 0.025987 | 0.387441 | –1.367950 |
| oxamic acid | 10.2087 | 171 | 3.429640 | 0.009827 | 2.65E+08 | 27.981225 |
| phosphate | 10.3103 | 158 | 5.607730 | 0.000002 | 0.109505 | –3.190938 |
| ribose-5-phosphate 1 | 19.5842 | 299 | 2.334980 | 0.028852 | 5.696613 | 2.510104 |
| Threitol | 13.3109 | 217 | 1.179820 | 0.020816 | 4.131765 | 2.046758 |
| tryptophan 1 | 20.7595 | 202 | 1.295940 | 0.039507 | 0.475206 | –1.073376 |
| xanthine | 18.9413 | 243 | 1.717660 | 0.001189 | 1.915535 | 0.937747 |

**Supplementary Table 3. Primers used in qRT-PCR.**

| Name | Sequence 5′-3′ | Application |
|---|---|---|
| Alas1 F | GGCTGTCATGGAGACTGTGAA | for *Alas1* gene |
| Alas1 R | GCATTGGTGGCAAGGAGGT | for *Alas1* gene |
| ces2a F | ACACAGCAGCACGGATGAT | for *ces2a* gene |
| ces2a R | GGCAGAGTCTTGGTCCAGAA | for *ces2a* gene |
| Cyp1a2 F | GCACTACCAAGACTTCAACAAGAA | for *Cyp1a2* gene |
| Cyp1a2 R | GCAGGATGGCTAAGAAGAGGAA | for *Cyp1a2* gene |
| Derl3 F | TTCTCTTCGGTGGTGTTCTTATGA | for *Derl3* gene |
| Derl3 R | ATGGAGGCATAGGCAATGTGAA | for *Derl3* gene |
| Fmo3 F | TCTGACTGGTGGTACACAAGG | for *Fmo3* gene |
| Fmo3 R | TGTCTGGATGGTGGTGCTATT | for *Fmo3* gene |
| prom1F | TAGCATCAGACCAAGCCTAAGC | for *prom1* gene |
| prom1R | TCATAAGGTTCGCAGAGCAACT | for *prom1* gene |
| GAPDH F | ACCACAGTCCATGCCATCAC | for control *GAPDH* gene |
| GAPDH R | TCCACCACCCTGTTGCTGTA | for control *GAPDH* gene |
